# Isolated Arthroscopic Lateral Retinacular Release for Lateral Patellar Compression Syndrome

**DOI:** 10.3390/life11040295

**Published:** 2021-03-30

**Authors:** Filippo Migliorini, Christian Lüring, Jörg Eschweiler, Alice Baroncini, Arne Driessen, Filippo Spiezia, Markus Tingart, Nicola Maffulli

**Affiliations:** 1 Department of Orthopedics, University Clinic Aachen, RWTH Aachen University Clinic, 52064 Aachen, Germany; migliorini.md@gmail.com (F.M.); Christian.Luering@klinikumdo.de (C.L.); joeschweiler@ukaachen.de (J.E.); alice.baroncini@gmail.com (A.B.); adriessen@ukaachen.de (A.D.); mtingart@ukaachen.de (M.T.); 2 Department of Orthopedics, Orthopedic Clinic Dortmund, 44137 Dortmund, Germany; 3 Department of Orthopedic and Trauma Surgery, San Carlo Hospital, 85100 Potenza, Italy; spieziafilippo@gmail.com; 4 Department of Medicine, Surgery and Dentistry, University of Salerno, Via S. Allende, 84081 Baronissi, Italy; 5 School of Pharmacy and Bioengineering, Keele University School of Medicine, Thornburrow Drive, Stoke on Trent, Keele ST5 5BG, UK; 6 Centre for Sports and Exercise Medicine, Barts and the London School of Medicine and Dentistry, Queen Mary University of London, Mile End Hospital, 275 Bancroft Road, London E1 4DG, UK

**Keywords:** knee, patellofemoral, lateral patellar compression syndrome, lateral retinacular release

## Abstract

Introduction: Evidence concerning the role of isolated lateral retinacular release (LRR) for lateral patellar compression syndrome (LPCS) dates back at least three decades. Appropriate indications, execution and outcomes still remain unclear and controversial. The present investigation analyzed the midterm result of isolated and arthroscopic LRR for LPCS in a cohort of patients who underwent such procedure at our institution. Material and methods: Patients undergoing isolated arthroscopic LRR for LPCS were identified retrospectively from our electronic database. All procedures were performed by two experienced surgeons. Patients with bony and/or soft tissues abnormalities, patellofemoral instability, moderate to severe chondral damage were not included. Patients with previous surgeries were not included, as were those who underwent combined interventions. Clinical scores and complications were recorded. Results: 31 patients were recruited in the present investigation. The mean follow-up was 86.0 ± 22.8 months. The mean age of the patients at the index operation was 34.2 ± 13.1 years. A total 55% (17 of 31) were women, and 58% (18 of 31) had involved the right knee. The mean hospitalization length was 3.5 ± 1.4 days. At a mean follow-up of 86.0 ± 22.8 months, the numeric rating scale (NRS) was 1.2 ± 0.8, the Kujala score was 91.3 ± 11.3, the Lysholm score was 93.1 ± 15.0, and the Tegner score was 5.0 ± 1.8. At the latest follow-up, 9 of 31 (29.0%) of patients experienced compilations. One patient (3.2%) had a post-operative hemarthrosis which was managed conservatively. Six patients (19.4%) reported a persistent sensation of instability, without signs of patellar dislocation or subluxation. One patient underwent an arthroscopic meniscectomy, and another patient an anterior cruciate ligament (ACL) reconstruction. Conclusion: isolated arthroscopic lateral retinacular release for lateral patellar compression syndrome is feasible and effective, achieving satisfying results at more than seven years following the procedure.

## 1. Introduction

Lateral patellar compression syndrome (LPCS) is a common cause of patellofemoral pain [1]. The etiology of LPCS is still not fully understood [2]. Clinically, patients with LPCS present shortened and tight lateral retinaculum, combined with lateral patellar tilt [3]. This causes patellar mal-tracking, venous engorgement in the patella, and overload of lateral compartment of the patellofemoral joint [4,5,6]. Possible consequences are chondral damage, anterior knee pain and, in the long-term, patellofemoral joint osteoarthritis [1,7]. The management of LPCS is still debated, and no evidenced-based guidelines are available. Merchant et al. in 1974 introduced lateral retinacular release (LRR) as treatment for LPCS [8]. This technique aims to remove the overload of the lateral articular facet, and restore patellar tracking.

The evidence concerning the role of isolated LRR for LPCS mostly arises from studies now dating several decades, and the surgical outcomes of isolated LRR remain unclear. The present study reports the midterm outcomes of a cohort of patients who underwent isolated LRR for LPCS at our institution.

## 2. Material and Methods

### 2.1. Study Design

The present study was conducted according to the Strengthening the Reporting of Observational Studies in Epidemiology: the STROBE Statement [9]. Patients undergoing isolated all inside arthroscopic assisted LRR for LPCS were identified from our institutional database. All the surgeries were performed in the period 2008 to 2014 at the Department of Orthopaedic Surgery of the RWTH University of Aachen, Germany, and of the San Carlo Hospital of Potenza, Italy. All procedures reported in the present investigation were approved by the Ethics Committee of the Medical Faculty of the RWTH University of Aachen, Germany (project ID EK 438-20). All patients were able to understand the nature of their treatment and provided written consent to use their clinical and imaging data for research purposes. This study has been conducted according to the principles expressed in the Declaration of Helsinki.

### 2.2. Eligibility Criteria

**Figure 1:**
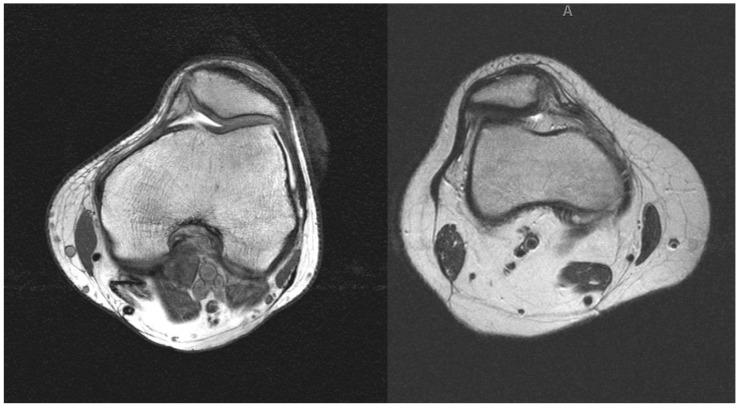
Evidence of lateral patellar compression syndrome (LPCS) at MRI.

All patients underwent plain long leg antero-posterior, knee lateral, and axial radiographs at 30 and 60 degrees (Ficat views) of knee flexion to evaluate Q-angle, patella alta, trochlear and patellar dysplasia. MRI was used to evaluated tibial tubercle—trochlear groove (TT-TG) and the chondral status of the trochlea and patella. The inclusion criteria were: (1) patellofemoral pain that affects the quality of life and participation to recreational activities; (2) failed previous conservative management and physiotherapy for at least 6 weeks; (3) MRI evidence (Figure 1); (4) same surgical technique, instrumentation and post-operative protocol; and (5) patients able to understand the nature of the treatment. Previous conservative therapies included non-steroidal anti-inflammatory drugs, cryotherapy, taping, braces, platelet-rich plasma infiltrations, and absence from recreational activities. Previous physiotherapy included stretching, leg and pelvic muscles reinforcement, closed and open chain exercises, massage, proprioceptive and postural exercises.

The exclusion criteria were: (1) any previous patellar dislocation or subluxation; (2) any previous knee surgical intervention; (3) lower limb mal-alignments and/or (4) deformities of the knee in the frontal plane; (5) patella baja or patella alta; (6) chondropathy; (7) any other condition that may influence the results of the present investigation (Table 1).

### 2.3. Surgical Technique

All procedures were performed by two experienced fellowship trained orthopedic surgeons. With the patient under epidural anesthesia and placed supine, a tourniquet was applied at the root of the thigh and inflated to 300 mm/Hg after exsanguination. Routine prepping of the operating field was performed. Conventional anterolateral and anteromedial arthroscopic portals were used [14]. The anterolateral portal was used for the inspection of the knee joint. An arthroscopic hook was used to examine the integrity of the menisci and cruciate ligaments. The cartilage was also inspected. Any hypertrophic inflammatory synovial tissue and synovial fold was shaved. Patellar tracking was examined bringing the knee through the full range of motion, to determine the location and extent of LPRR. With an electrocautery, LRR was performed from the proximal to the distal pole of patella, starting below the distal end of the vastus lateralis obliquus (VLO), close to the lateral aspect of the patella to avoid involvement of the iliotibial band. The soft tissues involved in the release were the capsule and the superficial and deep layer of the retinaculum. The extension of LRR was determined by the position of the patella. Satisfactory arthroscopic repositioning was considered to have been achieved when (1) the patellar sulcus was centered in the femoral trochlea and (2) the articular surfaces of the lateral patellar and trochlear compartments were symmetrically spaced. Subsequently, the patella was tested clinically. Satisfactory release was considered to have been achieved reached if medial patellar displacement of at least 12 mm and a patellar tilt of 90 degrees were possible. Further electrocautery ablation was used for hemostasis, followed by flushing the joint. A drain was kept in place for 48 h. Quadriceps muscle strength training started after 48 h. Patients were allowed to return to sport six weeks after the index procedure.

### 2.4. Outcomes of Interest

The following data were recorded: age at time of surgery, gender, side, hospitalization length. During the survey, patients were called by an assessor who had not been involved with the clinical management of the patients. The following scores were asked: numeric rating scale (NRS 0-10), Kujala Anterior Knee Pain Scale [15], Lysholm Knee Scoring Scale [16], and the Tegner Activity Scale [17]. Moreover, the following complications were recorded: anterior knee pain, persistent sensation of instability, further surgeries, and patella-related diseases. Sensation of persistent instability was defined as recurrence and/or subjective sensation of subluxation or instability [1].

## 3. Results

### 3.1. Search Results

**Figure 2:**
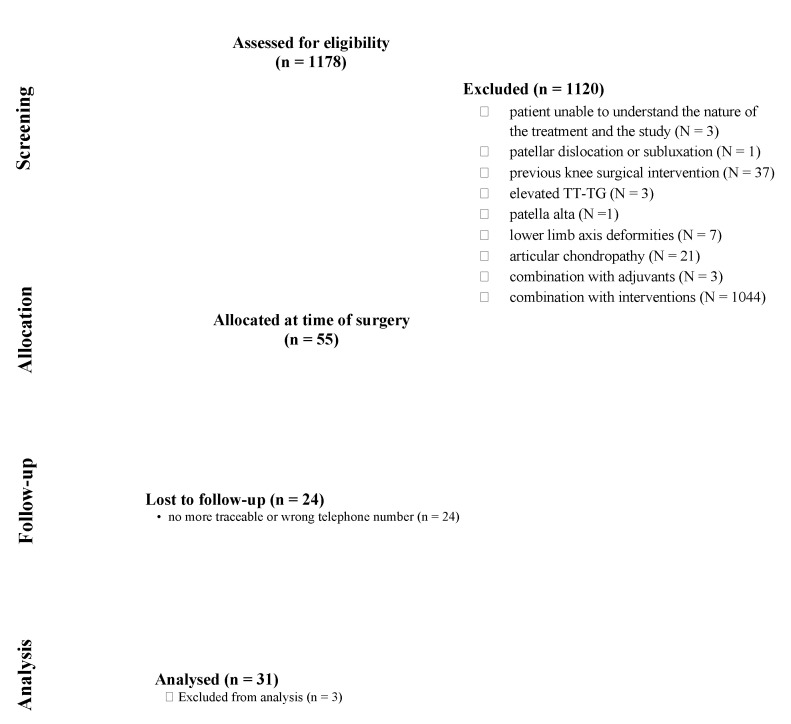
Diagram of the recruitment selection process.

The database search resulted in 1178 patients. A total of 1147 patients were excluded: patient unable to understand the nature of the treatment and the study (N = 3), previous patellar dislocation or subluxation (N = 1), previous knee surgical intervention (N = 37), elevated TT-TG (N = 3), patella alta (N =1), lower limb axis deformities (N = 7), articular chondropathy (N = 21), combination with adjuvants (N = 3), combination with other interventions (N = 1044), others (N = 3). A total of 55 patients were enrolled in the present investigation. Further 24 patients were no longer telephonically traceable. Finally, 31 patients were available to take part in the present investigation. The STROBE diagram of the recruitment is shown in Figure 2.

### 3.2. Patient Demographics

The mean length of the follow-up was 86.0 ± 22.8 months. The mean age of the patients at the index operation was 34.2 ± 13.1 years. 55% (17 of 31) were women, and 58% (18 of 31) had involved the right knee. The mean hospitalization length was 3.5 ± 1.4 days. Patient demographic is shown in Table 2.

### 3.3. Clinical Assessment

At a mean follow-up of 86.0 ± 22.8 months, the NRS was 1.2 ± 0.8, the Kujala score was 91.3 ± 11.3, the Lysholm score was 93.1 ± 15.0, and the Tegner score was 5.0 ± 1.8 (Table 3).

### 3.4. Complications

At the latest follow-up, 9 of 31 (29.0%) of patients experienced compilations. One patient (3.2%) had a post-operative hemarthrosis which was managed conservatively. Six patients (19.4%) reported a persistent sensation of instability, without signs of patellar dislocation or subluxation. One patient underwent an arthroscopic meniscectomy, and another patient an anterior cruciate ligament (ACL) reconstruction.

## 4. Discussion

The present study indicates that isolated lateral retinacular release is a reliable procedure in patients with lateral patellar compression syndrome, achieving satisfying results at approximately seven years follow-up.

In LPCS shortening and tightness of the lateral retinacular with an increased lateral patellar tilt as a result of hypertrophy of the lateral retinacular are present, without previous cartilage lesions or patellar instability [3]. It is necessary to differentiate LPCS from other causes of patellofemoral pain. Anatomical risk factors associated to patellofemoral instability must be considered [18,19,20]: analysis of the morphology of the trochlea and patella and lower limb mal-alignment is necessary [21]. A *Q* angle greater than 20 degrees is associated with worse surgical outcomes [22], and worse results are expected in patients with lower limb mal-alignment [23]. Additionally, patellar hypermobility has been considered as a negative prognostic factor [23]. MR imaging allows to ascertain the cartilage status [23]: the presence of chondropathy is a negative prognostic factor [22]. For advanced chondropathy, namely Outerbridge grade III to IV [2], LRR should be combined with lateral patella facetectomy [24]. Young women generally had worse outcomes at mid-term follow-up [25].

LRR is widely performed in combination with several interventions, especially with arthroplasty and distal or proximal realignments in patients with patellofemoral instability. However, the only indication for insolated LRR typically remains the LPCS [26,27]. In a recent study, arthroscopic LRR was performed in 11 athletes with isolated symptomatic Type III bipartite patella [28]. At a mean of 70 months, the authors found excellent functional outcomes and no complications. All the patients returned to sport after 42.3 days after surgery [28].

LRR can provide a relatively quick pain resolution for LPCS [21,22,23,29,30]. The decrease of articular high pressure restores patellar tracking, and preserves the surfaces from early degeneration. In addition, the release may denervate the patella and promoted amelioration of the pain [27,31]. Appropriate indications and technical expertise are important [32]. Hemarthrosis is a very common complications after LRR, as are iatrogenic medial patellar instability and quadriceps muscle weakening caused by an excessive tissue release [29,32,33,34,35]. Surgical identification and hemostasis of the superolateral genicular artery is important to reduce the incidence of hemarthrosis [20,32]. The risk of medial instability relates to the extent of the release. LRR reduces the force to laterally displace 10 mm the patella by 14–20% during the first 30 degrees of flexion [36]. To avoid medial instability, the release proximally should not go beyond the vastus lateralis obliquus (VLO) and careful intraoperative assessment of patellar tracking across the whole range of motion is necessary [33,34]. While longer release lead to medial instability, shorted releases lead to an unchanged clinical picture, and thus to failure [23,29,32,37,38]. Intra-operative skin burns and complex regional pain syndrome have been also described after LRR [39].

Even if LRR for LPCS is widely accepted, there are still several unresolved issues that must be addressed. Future studies should investigate and overcome current controversies, so as to delineate more rigorous eligibility criteria and clarify the best surgical procedures. The effect of different sites of the release along the lateral retinaculum, whether proximal or distal, and its effect on the patellofemoral biomechanics has been poorly investigated. LRR can be performed via a lateral parapatellar incision, a mini-open percutaneous approach, and arthroscopically [6,40,41], but it is still unclear which is more appropriate. Probably, open LRR allows better visualization of the anatomical structures, sparing the joint capsule and allowing the surgeon to identify the superior and inferior geniculate arteries, allowing direct accurate hemostasis. On the other hand, the arthroscopic technique allows direct visualization of the patella track during the whole range of motion. Although it is challenging, assessment of the soft-tissue balance between the medial and the lateral restraints is fundamental to achieve good clinical outcome and avoid complications [42]. Some authors advocated the use of Z-plasty lengthening of the lateral retinaculum to reduce the tension of the lateral tissue restraints and preserve the integrity of the soft tissues [43]. Moreover, to prevent over release, medial instability, and extensor mechanism weakness, lateral retinacular lengthening (LRL) has been advocated [44]. Two prospective studies found greater functional outcomes and return to sport in the LRL compared to the LRR [45,46]. However, the extensive release performed in the LRR group may have affected the outcomes.

This study has several limitations. The most important limitation is the lack of pre-operative clinical data to assess the functional improvement of patients after LRR. The retrospective design of the investigation leads to inevitable biases. The limited number of analyzed patients is also an important limitation. The unblinded nature of the study design, along with the lack of a control group, represent other important limitations. Finally, we highlight the high rate of patients lost at follow-up. These patients were relatively young at the time of the index procedure: although we kept careful notes of their place of residence and of their contact details, during the long period of follow up they would have moved away from their original address, changed their telephone number or email. A high rate of patients lost at follow-up in long term observational studies is unfortunately not uncommon. Given these limitations, results from the present study must be interpreted with caution. The highly standardized operative indications and post-operative protocols and rehabilitation program represent important strengths of the present study. Moreover, all procedures were performed by surgeons who were well beyond their learning curve. Further high-quality trials comparing LRR and other surgical and/or conservative strategies could be planned to demonstrate which one offers superior outcomes.

## 5. Conclusions

Isolated arthroscopic lateral retinacular release for lateral patellar compression syndrome is feasible and can be effective in patients without severe or evident patellar dysplasia, achieving satisfying results at approximately seven years follow-up.

## Figures and Tables

**Table 1:** Eligibility criteria (TT-TG: tibial tubercle—trochlear groove).

Eligibility Criteria
**Inclusion Criteria**
Patellofemoral pain that affects the quality of life and participation to recreational activities Failed previous conservative therapy and rehabilitation for at least 6 weeks MRI evidence of LPCS Same surgical technique, instrumentation and post-operative protocol Patient able to understand the nature of the treatment
**Exclusion Criteria**
Previous patellar dislocation or subluxation Any previous knee surgical intervention Limb mal-alignment: TT-TG distance > 20 mm Frontal plane lower limb deformities: 15° < *Q*-Angle > 22° Patella height: <0.8 Insall-Salvati ratio [10] > 1.2 Chondropathy: Outerbridge II to IV [11] Trochlea dysplasia Type B, C, D of Dejour [12] Patellar dysplasia Type III to IV of Wiberg [13]

**Table 2:** Demographic data of the patients.

Endpoint	Value
Procedures *(n)*	31
Age *(mean)*	34.2 ± 13.1
Sex (*female*)	55% (17 of 31)
Side (*right*)	58% (18 of 31)
Hospitalization length (*days*)	3.5 ± 1.4
Length of follow up (*months*)	86.0 ± 22.8

**Table 3:** Results of numeric rating system (NRS), Kujala, Lysholm and Tegner scores.

Scores	Value
Numeric Rating System	1.2 ± 0.8
Kujala Anterior Knee Pain Scale	91.3 ± 11.3
Lysholm Knee Scoring Scale	93.1 ± 15.0
Tegner Activity Scale	5.0 ± 1.8

## Data Availability

Data is contained within the article.
